# Assessing national action through emergency paid leave to mitigate the impact of COVID-19-related school closures on working families in 182 countries

**DOI:** 10.1177/14680181221123800

**Published:** 2022-09-12

**Authors:** Amy Raub, Jody Heymann

**Affiliations:** University of California, Los Angeles, USA; The University of Melbourne, Australia; University of California, Los Angeles, USA

**Keywords:** Childcare, COVID-19, education, global, paid leave, social protection, working families

## Abstract

In April 2020, nearly 1.6 billion learners were out of school. While a growing body of literature has documented the detrimental impact of these closures on children, less attention has been devoted to the steps countries took to mitigate the impact of these closures on working families. Paid leave is recognized as an important policy tool to enable working parents the time they need to respond to family needs without risking job or income loss. This article uses a novel data set to assess whether countries had policies in place prior to the pandemic to respond to increased care needs and the extent to which policies were introduced or expanded during the pandemic to fill the gap. Only 48 countries had policies in place prior to the pandemic that could be used to respond to the care needs created by school and childcare center closures. In the vast majority of these countries, the duration of leave in these policies was too short to meet the care needs of the pandemic or relied on parents reserving extended parental leave options. Only 36 countries passed new legislation during the pandemic, but the majority of those that did covered the full duration of closures. As countries continue to face COVID-19 and consider how to better prepare for the next pandemic, emergency childcare paid leave policies should be part of pandemic preparedness frameworks to prevent further exacerbating inequalities. The policies introduced during the pandemic offer a wide range of approaches for countries to identify feasible solutions.

In March 2020, as the world began to recognize the threat of the novel coronavirus 2019 (COVID-19), countries around the world began closing schools and childcare centers in an effort to contain and reduce disease spread (UNICEF, 2021). A growing body of literature has highlighted the negative impacts of these closures on educational attainment (Alban Conto et al., 2021; McCoy et al., 2021), children’s physical health (Goldfeld et al., 2022; Rahman and Chandrasekaran, 2021; Woolf et al., 2020), children’s mental health (Fong and Iarocci, 2020; Viner et al., 2022), caregivers’ physical and mental health (Almeida et al., 2020), and gender equality in employment (Carli, 2020). Less research attention has been paid to the policy options available to governments to lessen the impact of school closures on families. Utilizing a novel data set, this article presents a comparative analysis of country approaches to providing paid leave during these closures to understand the extent to which policies adequately supported care needs, how they were structured to support families during a time of economic crisis while still being economically feasible, whether they promoted gender equality in care roles, and whether there were barriers to accessing leave. These policies are critical to ensuring job and income support for working parents.^1^

## Impact of the COVID-19 pandemic on children and families

UNICEF (2021) estimates that during the first year of the COVID-19 pandemic, on average across countries, schools were closed for about half of the time that children would have normally been in school. Increased care needs from these closures had direct impacts on parental employment. In many settings, alternative care by older family members was forbidden or discouraged by public health officials due to the higher risk of mortality and morbidity for older caregivers (Cantillon et al., 2021). With the transition to remote learning, parents took on increased time supporting their children’s education (Bansak and Starr, 2021), in addition to time needed to provide direct care. Increased care responsibilities of the pandemic primarily impacted women’s employment. Across countries, women were 1.8 times more likely than men to report employment loss due to increased care needs in March 2020 with this gender gap widening to 2.4 in September 2021 (Flor et al., 2022). A study of parent’ employment in the United States found that the loss of full-time childcare due to childcare closures was associated with a loss of employment for mothers, but not fathers (Petts et al., 2021). A more rigorous study of employment outcomes in Canada using a triple difference approach found that the re-opening of primary schools had a positive impact on whether parents were employed and at work for both mothers and fathers, but that the impact was greatest for single mothers (Beauregard et al., 2022).

Impacts on parental employment were also seen in countries where multigenerational families are more common. One study in Kenya estimated that school closures contributed to 30% of the pandemic decline in work hours, at a cost of more than 3% of GDP (Biscaye et al., 2022). Another study examining the impact of the lockdown in South Africa, which included school and childcare center closures, suggests that the increased childcare burden of these closures contributed to women’s relatively higher reduction in employment and work hours compared to men (Casale and Posel, 2020). These findings are consistent with reviews of studies that show the impact of childcare availability and affordability on maternal employment outcomes in high- and low-income settings alike (Akgunduz and Plantenga, 2018; Halim et al., 2021; Harper et al., 2017; Morrissey, 2017). These findings are also unsurprising given the large number of families who are separated geographically from extended families. Moreover, even when older relatives do live nearby or within the same household, they may be unavailable to provide care because they are employed themselves or may have health problems that make them care recipients instead of caregivers (Heymann, 2006).

## Short-term school closures as a public health tool

While the detrimental impacts of school closures on children and families are clear, and there is ample reason to hope that policymakers in the future will find ways to prioritize children’s continued education while protecting public health, there may still be a need for short-term closures as new viruses or variants emerge. Understanding policy approaches that can help to mitigate some of the employment and economic impacts of school closures is important to enable public health officials time to find ways to address safety in schools. Moreover, school closures are not a new public health tool and have been used in previous epidemics, such as Ebola, SARS, and influenza, including H1N1, to contain disease spread (Fong and Iarocci, 2020; Smith, 2021). Studies of school closures related to influenza in the United States, Australia, and Japan provide examples of short-term school closures that have been used during severe outbreaks. During these shorter closures, parents needed to miss work to provide care in between 13% and 45% of households studied (Borse et al., 2011; Effler et al., 2010; Gift et al., 2010; Mizumoto et al., 2013; Timperio et al., 2009).^2^ Across studies, parents were more likely to miss work when all adults in the household worked or children were younger and needed more supervision. Indeed, the only study identified that did not show an impact on parental employment during school closures had both a majority of households with an available caregiver (76%) and a median age of children of 12 years old (Johnson et al., 2008).

## Income support during absences from work as a policy approach

Income support for parents who need to take time away from work to care for their children has been identified as a policy solution to help parents balance care responsibilities during school and childcare center closures while maintaining income security and employment (Gromada et al., 2020; Mizumoto et al., 2013; Zheteyeva et al., 2017). Ensuring economic stability for families, especially those already living month to month, and preventing parental job loss, particularly during times of economic downturn, matters to children’s health and educational outcomes in the short and long-term (Gregg et al., 2012; Mari and Keizer, 2021; Ruiz-Valenzuela, 2020; Sigurdsen et al., 2011).

In the absence of income support or paid leave, children are at increased risk of being left with inadequate supervision or with less support for remote learning. Previous research suggests that caregivers do not make these decisions lightly, but that when faced with an impossible choice between a family’s economic survival and children’s adequate supervision, children may be left at home alone (Ruiz-Casares and Heymann, 2009). In the context of the pandemic, parents may also be present in the household, but unable to provide the support needed for online schooling. Studies from the United States and Germany estimate that parents spent on average between 2.5 and 3 hours per day supporting children during remote learning (Carpenter and Dunn, 2020; May et al., 2021). Income support and paid leave can help support parents’ role in children’s education.

Beyond the job and income security provided by paid leave policies, paid leave can also support caregiver mental health. For example, a study of family caregivers in the United States found that access to paid leave was associated with a higher mental and physical health score (Earle and Heymann, 2011). Similarly, a study of parents of children with special health needs found parents receiving full pay while on leave were 1.7 times more likely to report positive effects on their own emotional health than parents taking unpaid leave (Schuster et al., 2009). In the midst of the pandemic, mechanisms to reduce parental stress are particularly important given the high levels reported in countries around the world (Almeida et al., 2020). Moreover, the unique nature of parental stress during the pandemic brought on by isolation from peers and more time spent together in close quarters compounded by job loss and economic insecurity have been identified as risk factors for child maltreatment during a time when mechanisms to prevent and intervene were more limited (Goldfeld et al., 2022; Rodriguez et al., 2020).

Finally, income support and paid leave disproportionately matters to women’s employment and economic opportunities. Women remain primary caregivers in countries around the world with consequences for economic equality (International Labor Organization (ILO), 2018). Paid parental leave has been shown in high-income countries to support women’s economic opportunities by supporting women’s continued attachment to the labor force after the birth of a child (Nandi et al., 2018). Moreover, paid leave policies that are structured in a way to support men’s engagement in caregiving can help to shift discriminatory norms that perpetuate gender inequality (Omidakhsh et al., 2020). While there has been less rigorous research on the impact of paid leave in lower income countries and one associational study found no relationship between paid maternity leave and female labor force participation likely due to already high employment rates for women (Fallon et al., 2017), at least one study suggests that paid maternity leave is associated with lower rates of gender occupational segregation (Chang, 2004). More research is needed to fill this gap in the literature. In the context of school closures, caregiving needs may impact not only working mothers but also older girls who become responsible for care of younger siblings (Burzynska and Contreras, 2020). Given the increasing recognition of the importance of gender equality for children, health, and national economies (Darmstadt et al., 2019; Madgavkar et al., 2020), polices that support gender equality in the midst of the pandemic are particularly important.

## Measuring national action on paid leave

Despite the importance of income support and paid leave to enabling working parents to provide care and educational support for children during the school and childcare center closures associated with the COVID-19 pandemic, little is known about the approaches countries have taken to providing paid leave. The World Bank Group’s (2021)*Women, Business and the Law* provided a first look at the number of countries that had passed paid leave policies to respond to these closures. However, a more in-depth analysis is needed. This article uses a novel data set to assess the structure and adequacy of paid leave for policy changes that occurred during the pandemic, as well as whether there were any policies in place prior to the pandemic to meet these caregiving needs.

## Methods

### Data source

To assess the adequacy of paid leave policies in place during the pandemic, two databases of paid leave policies were constructed, transforming policy features into categorical and numerical variables.^3^ The first database measured emergency measures that countries took during the pandemic to provide paid leave to working parents. The second database assessed policies in place prior to the pandemic that could be used to meet emergency childcare needs during the pandemic.

To construct the measures of paid leave policies introduced or expanded during the pandemic, a multilingual team of policy analysts fluent in English, French, Spanish, and other languages leveraged existing COVID-19 policy measure trackers in place as of 1 September 2020 to identify which countries introduced measures between March and August 2020. We focused on the first 6 months of the pandemic for two reasons. First, this period captured rapid responses to the unfolding public health and economic crisis. Second, this period of time covers the peak of school closures globally (UN, 2020).

For all UN member states, policy analysts checked the OECD Government Social Policy Responses and Tax Policy Response database, the ILO Social Protection Responses database, and the Eurofound Policy Watch Database. If no relevant policy was found using these sources, analysts additionally checked the IMF’s Policy Responses to COVID-19, the World Bank Social Protection and Jobs Responses to COVID-19, and ISSA’s Coronavirus Country Measures Table. Across these sources, policy information was available for 182 of 193 UN member states.^4^

Once a relevant policy was identified, analysts located the original legislation through links in those data sets or government websites when working links were unavailable. In the rare cases,^5^ when no original legislation was available, analysts coded from official government websites or policy descriptions available through policy trackers, noting when there was insufficient data to determine all aspects of a policy. For each country, two policy analysts independently sourced and read the sourced text in full to answer questions about key policy features. To capture policies that would apply to all workers regardless of geographic location, only national level policies were coded. Because some countries have more generous employment policies for public sector workers than private sector workers, we similarly only coded policies that applied to private sector workers when approaches differed across sectors. Analysts then reconciled their answers and brought any remaining questions to meetings of the full coding team to be resolved. Once data coding was complete, systematic quality checks and reviews of outliers were conducted. Policy data were further cross-checked against newly available information that was published by the World Bank’s Women, Business, and the Law, the International Network on Leave Policies and Research, and the International Labor Organization’s NATLEX database (full check of all new legislation from 2020 available online as of July 2021).

To assess the availability of paid leave in place before the pandemic, labor codes and other relevant pieces of legislation were used to identify paid childcare leaves and other forms of leave that could be used to meet child caregiving needs available to private sector workers as of March 2019. When policies varied sub nationally, the least generous policy available to workers in the country was captured. Legislation was identified primarily through the ILO’s NATLEX database and coding methods were similar to coding for emergency paid leave policies.

### Variables

#### Types of paid leave

For paid leave available before the pandemic, we distinguished between three types of leave. First, extended or delayed parental leave and single childcare leave entitlement. In some countries, parents can opt to save a portion of their parental leave to use when their child is older (beyond age 3). In other countries, this is a set amount of leave available to parents for childcare needs until a child turns a certain age. Second, paid leave available to workers each year for casual, discretionary, or emergency reasons. Third, paid leave available to workers each year for family needs or family emergencies generally or childcare needs or school or childcare center closures specifically.

For paid leave available during the pandemic, we limited our analysis to policies that would provide income support while maintaining parents’ attachment to the labor force. We captured policies that guaranteed paid leave for childcare, provided payments directly to parents while they took leave and received no compensation from their employers, or provided subsidies to businesses that provided employees with paid leave for childcare needs. We did not include means-tested or universal payments to families with children or paid leave policies that narrowly applied to when children needed to quarantine due to testing positive or being exposed to COVID-19.

#### Duration of leave

To compare the duration of paid leave available across countries, we converted the duration stated in the original source to weeks using standard conversion factors of 7 calendar days per week, 5 or 6 working days per week depending on the country’s standard work week, and 4.3 weeks in a month.

#### Wage replacement rate

We examine three measures of the generosity of paid leave across countries. First, we compared the stated wage replacement rate across countries. Our measure is the general wage replacement rate not including supplements for single parents or children with disabilities. For countries where all families received a flat rate or fixed payment amount that was not tied to previous wages, we recorded the wage replacement rate as being paid at a flat rate. When wage replacement rates varied over the duration of leave, we recorded the rate for the initial period of leave.

Then, we calculated the payment that a worker earning minimum and average wages would receive. When workers received a flat rate payment, we divided this payment by the average wage to determine the effective wage replacement rate. When workers received a percentage of their previous earnings, we examined whether there were any caps on benefits that would reduce the effective wage replacement rate for a worker. For example, if the wage replacement rate was 80%, but benefits were capped at US$500 per month, a worker earning US$1000 per month would only receive US$500. We then divided the calculated benefit amount by the average wage to determine the percentage of average wages an individual would receive. In this example, an average wage earner would only receive 50% of wages due to the cap on payments. Gross monthly minimum wages for 2020 were obtained from ILOSTAT.^6^ Average wages were obtained from OECD Statistics supplemented with data from national statistical agencies.

#### Funding of leave

Many businesses faced economic challenges during the pandemic as lockdowns and the economic crisis impacted consumer behavior. Ensuring paid leave to support workers did not add additional direct financial costs to businesses is thus very important to the feasibility of these measures and to prevent some forms of discrimination and retaliation against working parents. To better understand whether countries mitigated the direct costs of paid leave to businesses, we captured how countries funded the leave, distinguishing between employer-provided paid leave, government-funded paid leave, and systems that relied on payments from both employers and government.

#### Barriers and supports to caregiving across gender and family type

To understand whether policies created barriers or supported caregiving across gender and family type, we examined three aspects of policy. First, was it gender restrictive? Second, where there were any provisions that encouraged both parents to take leave? Research from paid leave for children’s health needs suggests that men may be less likely to take paid leave when it is structured as a shared family entitlement (Boye, 2015; Eriksson, 2011). Third, did policies consider the additional time needs of single parents caring for children alone during closures compared to two-parent families?

#### Barriers to accessing leave

To understand whether policies created barriers to accessing leave, we examined eligibility criteria for leave and whether employers were required to guarantee leave requests.

### Analysis

All analyses were conducted using Stata MP 14.2. Differences were assessed by country income group using the Pearson’s chi-square statistics. Pearson’s chi-square is commonly used to assess whether differences in categorical data are statistically significant across two or more independent groups. Country income level was categorized according to the World Bank’s country and lending groups as of 2020.

## Results

### Paid leave available before the pandemic

**Figure 1.**
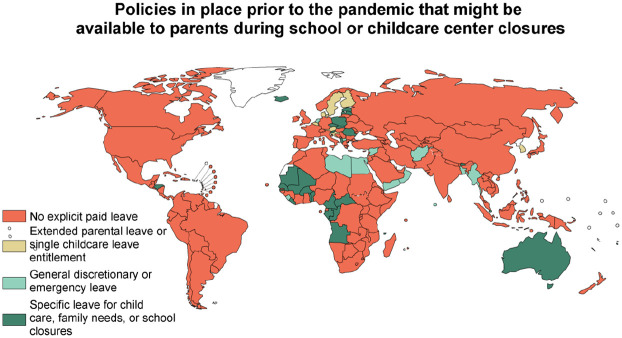
Prior to the pandemic, few countries have policies in place guaranteeing parents access to paid leave that could be used during school or childcare center closures.

**Figure 2.**
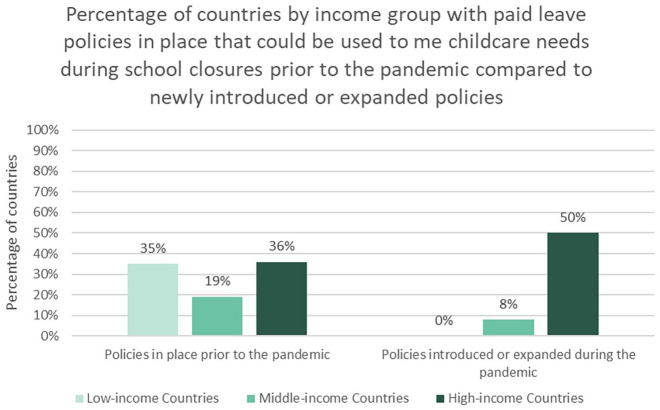
While countries across income groups had policies in place prior to the pandemic that could be used to meet childcare needs during school closures, primarily high-income countries introduced or expanded paid leave to meet emergency childcare needs during the pandemic.

Prior to the pandemic, only 48 countries took an approach to ensuring paid leave that could be used to support parental caregiving during a school or childcare center closure (Figure 1). These policies were equally common in low- and high-income countries (35% and 36% of countries, respectively), but slightly less common in middle-income countries (19%, *p* < 0.05 compared to high-income) (Figure 2).

In eight countries – all high-income – parents had access to extended paid parental leave or a single childcare leave entitlement that could be taken beyond early childhood to meet long-term care needs.^7^ The extent to which this paid leave would be available to parents during the pandemic in the absence of other legislative changes depends on the extent to which parents had already used this leave to support part-time transitions back to work, care during school holidays, or other care and work transitions. Other types of leave were available on an annual basis and distributed more evenly by country income. While these other forms of leave would be available to families every year, they were much shorter than the leave needs experienced by many parents during the pandemic: only three countries provided more than 2 weeks of paid leave for childcare, family needs, or emergencies on an annual basis.^8^

### Duration of paid leave policies introduced during the pandemic

**Figure 3.**
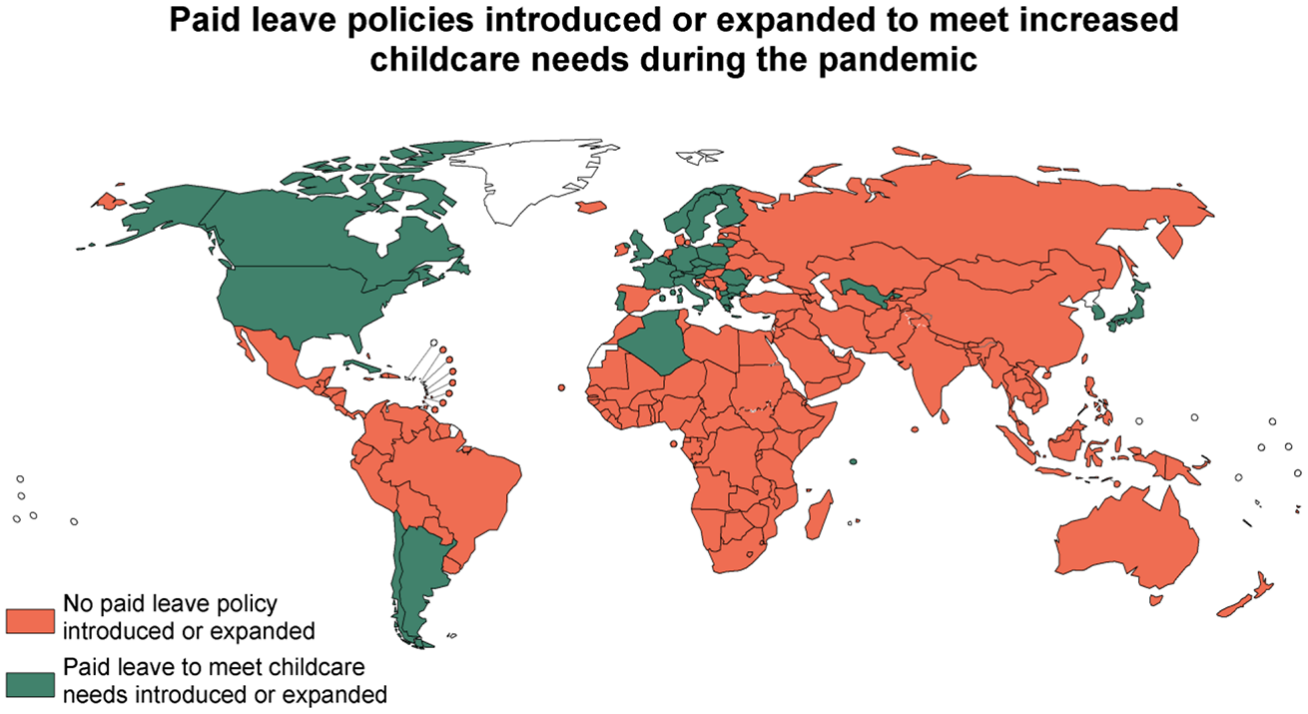
A minority of countries introduced or expanded paid leave to meet increased childcare needs during the first year of the pandemic.

In 2020, 36 countries were identified that introduced or expanded paid leave policies to enable parents to meet the increased care needs associated with school or childcare center closures (Figure 3). High-income countries were far more likely to provide support than middle-income countries (50% compared to 8%, *p* < 0.01) (Figure 2). No low-income countries were identified with new policies.

**Figure 4.**
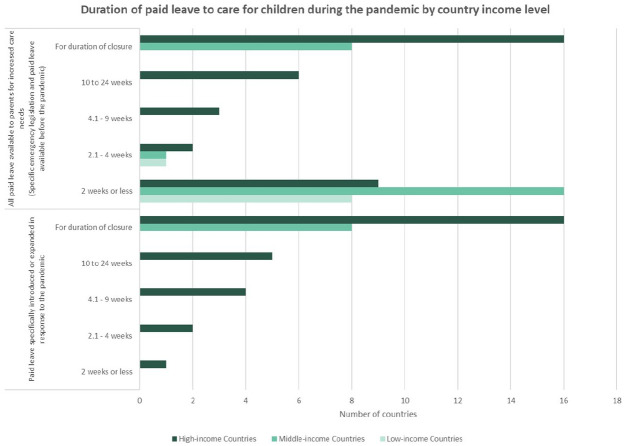
Most countries made paid leave available for the duration of school closures.

The most common approach for paid leave introduced during the pandemic was to make leave available for the full duration of the closure of school and childcare centers (Figure 4). All middle-income countries and the majority of high-income countries took this approach to legislating paid leave during closures. One country (Bulgaria) did not establish a limit on the maximum amount of paid leave, but provided a one-time payment to low-income families who had taken at least 20 days of unpaid leave. Legislation in place prior to the pandemic allows each parent to take up to 6 months of unpaid caregiving leave. Greece also did not establish an explicit limit on the duration of paid leave taken, but required that for every four days of leave taken, 1 day was deducted from parent’s annual leave entitlements.

Among countries that set a limit on the duration of paid leave, the shortest amount of leave was 2 weeks (Republic of Korea)^9^ and the longest length was 24 weeks (Canada and Sweden).^10^ Of the 12 countries that established a limit on the duration of paid leave, 10 countries made this leave available as a separate entitlement. In Canada, the paid leave for childcare during school closures was part of the Canada Emergency Response Benefit program that also covered periods of unemployment, worker’s personal illness, and caring for ill family members. In the United States, the entitlement of 12 weeks of paid leave for childcare was shared with paid leave for workers’ personal and family health needs.

Twelve countries that had policies in place prior to the pandemic that could be used to provide childcare also passed emergency legislation during the pandemic to respond to increased care needs. In six of these countries, a new policy was introduced to cover care needs during the pandemic. Three countries amended separate paid sick leave policies to cover care of healthy children during closures, and two countries amended the conditions of existing childcare leave policies to cover the full duration of closures. Just one country (Poland) increased the duration of existing childcare leave by a fixed amount. Prior to the pandemic, Poland allowed parents to take up to 60 days of paid leave to care for children during school or childcare center closures. During the pandemic, this was iteratively expanded to add 28 days to the existing entitlement by July 2020. In contrast, one country decreased paid leave entitlements for parents in the midst of the pandemic: Fiji reduced the duration of family care leave from 5 days to 2 days.

### Wage replacement rates of paid leave introduced during the pandemic

**Figure 5.**
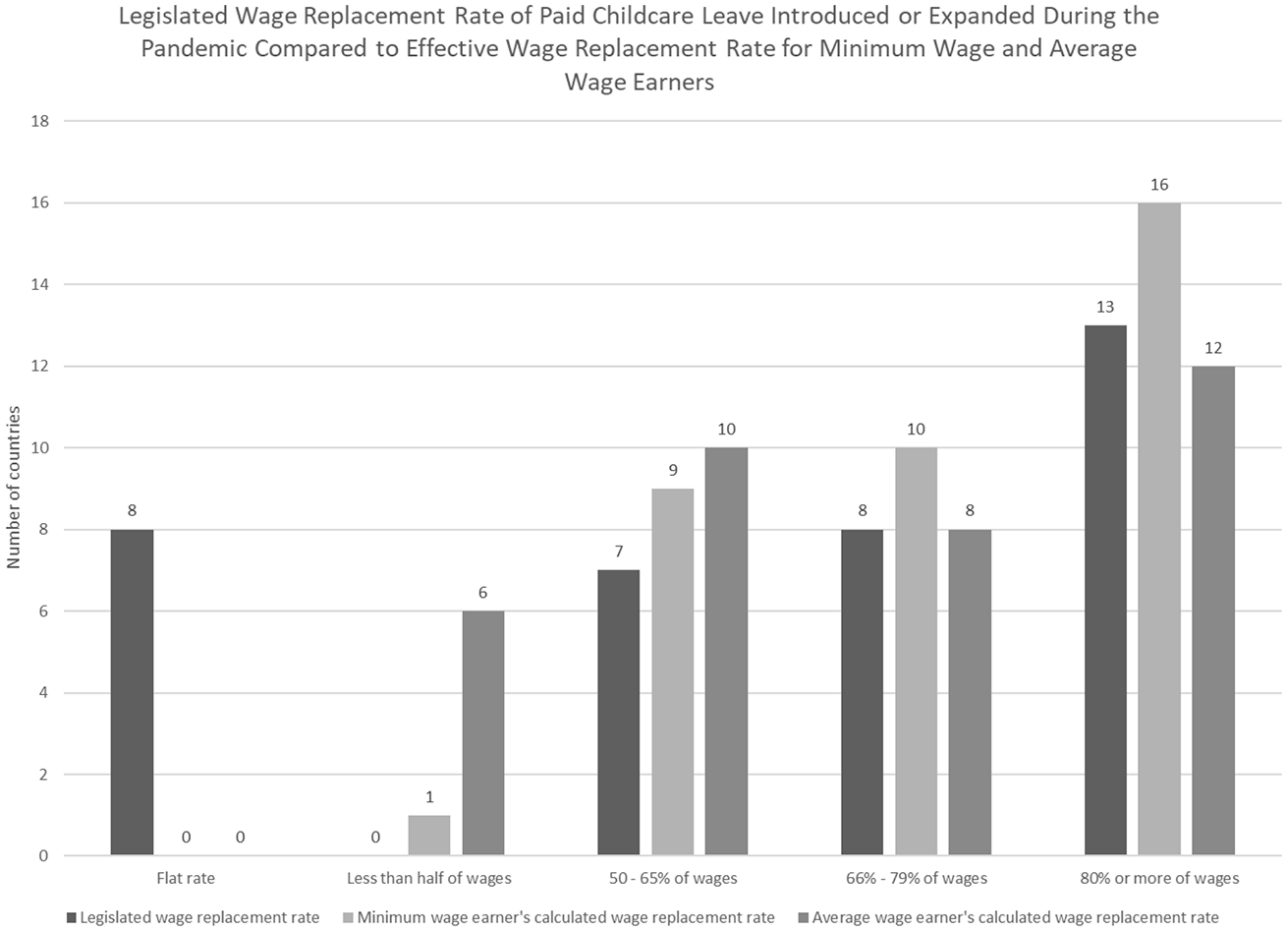
Legislated payment levels for paid leave were often low, impacting affordability to take leave.

Wage replacement rates for paid leave due to closures varied greatly, but were frequently low (Figure 5). Eight countries guaranteed parents a flat rate payment that was not tied to earnings. In four countries, these flat rate payments were so low that a parent earning minimum wage would receive less than two-thirds of their earnings while on leave which may make leave taking unaffordable for families. Only 13 countries guaranteed full wages or close to full wages. In two countries (Chile and Cuba), benefits were higher in the first month of leave than in subsequent months.

When considering the level of flat rate payments compared to average earnings and earnings-related caps on benefits, six countries guaranteed parents less than half of their previous earnings while on leave. Lower payments rates may make it more challenging for household finances for higher earning spouses to take leave.

Of the three countries that guaranteed 4 weeks or less of paid leave, two countries provided full wage replacement and one country (Korea) provided a flat payment that was roughly a third of wages for an average earning worker (results not shown). In contrast, only 9 of the 29 countries that guaranteed 10 weeks or more of leave or set no explicit limit on the duration of leave guaranteed full wages or nearly full wages.

While leave policies in place prior to the pandemic were generally short, nearly all of those available specifically for child care, family needs, or school closures were highly paid: 25 of 27 countries guaranteed workers full or close to full wages while on leave. The only countries to provide lower wage replacement rates were the Czech Republic (60%) and Slovakia (55%). Of note, when expanding the paid leave policy to meet the increased care needs of the pandemic, the Czech Republic also increased wage replacement rates to 80% in April 2020. In contrast, extended paid parental leave options that might have been available to parents during the pandemic if they had not previously exhausted it were all low paid, either as a low flat rate payment for all families or with a low cap on wage replacement rates that meant average wage earners would receive much less than full wages.

### Funding of paid leave

The majority of paid leave provisions were funded entirely through social security systems or public funds, reducing the burden on businesses during a time of economic crisis. In five countries, employers and government shared responsibility for funding leave with employers being responsible for half or less of leave costs (see Table 1 for descriptions). In three countries (Algeria, Macedonia, and Seychelles), employers were made directly responsible for paid leave benefits without a clear mechanism for reimbursement.

**Table 1.** Approaches to sharing responsibility between employers and government for paid emergency leave in countries that where both employers and governments financed paid leave.

Country	Approach to Funding Paid Leave
Austria	Employers could be reimbursed for a third of wages paid.
Greece	A quarter of paid leave came out of existing employer-provided annual leave entitlements and the remaining was financed 2/3 by social security and 1/3 by employers.
Monaco	Employers were encouraged to top up payments from the daily allowance they received from the insurance fund.
Portugal	Financing was jointly split between employers and social security.
Romania	Employers were reimbursed by the government for wages paid directly to the parents, but were still responsible for paying workers’ taxes and social security contributions while they were on leave.

In contrast, the majority of paid leave specifically for child care, family needs, or school closures available prior to the pandemic on an annual basis was funded by employers. Just 4 of 27 countries financed these leaves through social security or other government funds. In contrast, all of the extended paid parental leave options that might have been available to parents during the pandemic if they had not previously exhausted it were funded through social security systems.

### Barriers and supports for equal caregiving

While in most countries (34) leave entitlements were gender neutral, two countries had explicit barriers to men accessing paid leave. In Algeria, women with children were prioritized for accessing paid exceptional leave during the economic shutdown, but no such prioritization was given to men with children. In Cuba, the initial paid leave policy introduced in early April made paid leave only available to mothers.

While most countries had no specific provisions that would actively support both parents’ engagement in caregiving in two-parent households, a few countries took specific measures that might be likely to support sharing of leave taking. For example, in Belgium paid leave was only able to be taken part-time (either half of one-fifth of working hours) for two-parent families. Only single parents or parents of children with disabilities were able to take full-time leave. In seven countries, there were explicit provisions that allowed parents to alternate taking leave. For example, Andorra allowed both parents to take leave in shifts of full working days. While Czech Republic allowed parents to alternate taking leave to enable both parents’ involvement, prior to the pandemic each parent could take just nine consecutive days of leave, but during the pandemic, there was no limit on the number of days an individual parent could take.

In the majority of countries, leave entitlements covered the full duration of closures, so single parents would not necessarily need additional entitlements to the duration of paid leave. However, only 2 of the 11 countries that limited the duration of paid leave recognized the increased care burden for single parents by providing more leave than for individual parents in two-parent households. In Germany and Norway, single parents were entitled to twice as much paid leave as each parent in a two-parent household.^11^ Three countries guaranteed higher payment levels to single parents compared to two-parent households.

### Barriers to accessing leave

In some countries, restrictions on which employees were eligible for paid leave created barriers. The United States was alone in exempting both very large employers (500 or more employees) and allowing for exemptions for small employers (less than 50 employees). Two countries had exceptions for essential workers. Argentina excluded essential workers from the paid leave scheme and Romania required employer consent for workers in essential industries to take paid leave. An additional four countries more broadly required employer consent for employees to access paid leave or structured the program as a subsidy for businesses who gave paid leave to their employees during the pandemic.

In providing paid leave, countries commonly required that parents were unable to simultaneously work and provide care for their child while separately encouraging employers to make remote and flexible work options available to parents. Five countries, however, had stricter requirements that denied paid leave to parents who had the option to work remotely. In contrast, some countries recognized that teleworking may not provide sufficient time for parents to meet all care needs. For example, the United States’ Emergency Paid Sick Leave Act provided time for when an employee is ‘unable to work (or telework)’.

Countries also varied as to the level of school closure needed to qualify parents for leave. For example, in the United States schools were required to be closed for at least five consecutive days for parents to qualify. In contrast, parents in Cuba could access paid leave during school closures, as well as when schools were open but parents decided not to send their children.

Finally, while countries generally considered whether another adult was able to provide care during closures, some countries also considered the health of caregivers when determining eligibility for paid leave. In Switzerland, parents generally could not take paid leave during school holidays, but were eligible for leave if their children would normally have been cared for during the holidays by a vulnerable person. Andorra required parents to certify that neither had close relatives who could ‘reasonably’ take care of the children before being able to access paid leave, but broadly considered grandparents to be ‘unable to care for children’.

## Discussion

Prior to the pandemic, only 48 countries took an approach to ensuring a form of paid leave that could be used to support parental caregiving during a school or childcare center closure. The majority of policies that existed were too short for the scale of school closures triggered by the pandemic. Despite the urgent need for more support, only 36 countries supported working parents who needed to balance work and care responsibilities by passing or expanding paid leave policies to meet care needs during these closures.

These new and expanded policies demonstrate the feasibility of targeting relief to working parents to simultaneously support children’s healthy development alongside job and income stability for their parents. The majority of countries that enacted new measures did so for the duration of closures. In all but three countries, financing for these paid leave policies came in full or in part from social security or other government funds, reducing the direct costs to employers. While wage replacement rates were often low, 12 countries ensured workers earning average wages would receive 80% or more of wages while on leave and 16 countries did so for workers earning minimum wage.

At the same time, the gaps in the availability of these policies are striking. In April of 2020, more than ten percent of global GDP was being spent to respond to COVID-19 and nearly 1.6 billion children had their education interrupted (Chen et al., 2021; UN, 2020). Yet, 110 countries had no policies in place to ensure working parents could take care of their children during school or childcare center closures while maintaining job and income security.

Fifteen countries that passed new legislation during the pandemic to address paid leave for school closures did so by expanding existing policies. Providing a foundation to build on during a time of crisis ensures that important decisions, such as who is eligible, how it is financed, and how it will meet the needs of different family types, are made when there is more time to identify and agree upon solutions. All countries should consider having a permanent policy in place that can be used specifically in times of public health crisis as part of their pandemic preparedness response. These policies should be designed to cover the full duration of the closures. With planning, financing can be done in a way that minimizes the direct cost to businesses that may be strained during crises. Future research should also look at how past welfare state models affected policy formulation during the pandemic and the adequacy of these responses.

The decision to close schools in the face of disasters should always be weighed against the impact on children, particularly for marginalized children. Embedding paid leave for closures into disaster preparation frameworks will provide two additional benefits for governments. First, there will be additional known direct costs to governments when the decision to close schools or childcare centers is made. Ensuring responsibility for these costs will help to avoid unnecessarily lengthy closures that jeopardize children’s education. Second, supporting parents to provide nurturing care during this time will help to mitigate some of the impacts of closures on children. While this is certainly not enough to replace the lost learning or socialization time, providing families with equal opportunities to support their children during challenging times is fundamental to avoiding exacerbating disparities.

While half of high-income countries introduced or expanded emergency childcare paid leave policies during the pandemic, only a minority of middle-income countries and no low-income countries did so. This raises the question of whether these policies are only feasible and useful in higher income countries. Examining other similar types of leaves suggests these types of social protections do have the potential to be both feasible and useful. For example, paid sick leave is essential during a pandemic, enabling workers to stay home when sick while maintaining job and income security. We previously found that nearly all low-income countries have policies that guarantee paid sick leave from the first day of illness (Heymann et al., 2020), illustrating the feasibility of adopting these social protections. This is similarly true with paid leave for new mothers with 186 countries providing this leave (Heymann et al., Forthcoming).

Furthermore, research has also demonstrated that social protections for working parents make a difference in low- and middle-income countries. Rigorous quasi-experimental methods have been used to show paid maternal leave is causally associated with lower infant mortality in low- and middle-income countries as well (Nandi et al., 2016). Moreover, additional studies have illuminated mechanisms for reduced mortality in low- and middle-income countries through these social protections contributing to higher vaccination rates (Hajizadeh et al., 2015), increased breastfeeding duration (Chai et al., 2018), and lower rates of diarrheal disease for young children (Chai et al., 2020).

The feasibility and usefulness of paid leave policies in lower income settings does not mean that ensuring that economic support reach all parents is simple. There is value in understanding approaches that work for the formal economy even in settings where these policies cover a minority of workers. In many countries, enough households may have at least one family member working in the formal economy for these policies to have impact. Moreover, being able to retain these high-quality formal jobs in the midst of economic crisis is especially critical for women’s economic opportunities. Policies covering the formal economy can also have important consequences across the income spectrum through impacting approaches in the informal economy. For example, a study of the impact of increasing minimum wage in 23 low- and middle-income countries found impacts for child nutritional outcomes in even the poorest households (Ponce et al., 2018). Future research should examine more deeply whether and how approaches to income protection during the pandemic reached workers in the informal economy.

Yet, short-term leave policies are also the most challenging to get right for the informal economy. While other paid leave policies may effectively function as cash transfers, whether after the birth of a child or during times of serious illness, the administrative challenges of mobilizing payments rapidly to families are large. Future research should also consider complementary policies that were in place to support families, such as approaches to ensuring housing and food security. Finally, closures should be limited to short term due to the high cost to both children and economies of closing schools, particularly in settings where living conditions already facilitate rapid disease spread (Brewer et al., 2021).

Careful attention should also be paid to policy design decisions and eligibility conditions that can exacerbate or reduce disparities within countries. Parents should not be denied access to paid leave or income support because of their type of employment or the size of their employer. Nor should parents be expected to be able to simultaneously work and care for young children, whether these be workers in markets or teleworkers. More research is needed to understand the best approaches to ensuring that these increased care responsibilities do not derail women’s economic opportunities, but promising models have been identified that encourage both parents to take leave. Future studies should also examine implementation and the extent to which parents were aware of their legal rights and were able to exercise those rights without fear of retaliation.

On-going monitoring of the steps governments are taking to prepare for the next pandemic is important for supporting legal change. Ten years ago, the World Health Organization adopted the Pandemic Influenza Preparedness Framework focused on ensuring adequate surveillance and vaccine sharing for pandemics. Similar frameworks and monitoring should be adopted to support the social and economic consequences of pandemics. Mitigating the long-term effects of the pandemic on children through increased risk of childhood poverty, inadequate supervision and support for learning, and increased care responsibilities for older girls with less time for their own learning is fundamental to internationally agreed upon goals of reducing inequalities.

As countries begin to ‘build back better’, from the pandemic, it is critical that countries are better prepared for the next pandemic from an economic as well as public health standpoint. School closures had devastating impacts on children around the world. Governments can help support families during pandemics by ensuring parents can provide adequate care for their children while maintaining jobs and income security. Moreover, the policy choices that governments make matter to inequalities. Emergency childcare paid leave policies should become a part of every nation’s disaster preparedness framework.

## Notes

1. Throughout, we refer to working parents as parents who are working for pay outside the household. We include in this both parents who are working in the formal and informal economy. However, as noted in the methods, policies may have differential coverage across these groups.

2. Studies focused on the impact of school closures on parental employment were not identified outside the OECD. More research is needed to understand these impacts in different settings.

3. Data are available for download at worldpolicycenter.org.

4. Data were unavailable for the Democratic People’s Republic of Korea. Ten UN member states had no recorded cases of COVID-19 as of 1 September 2020 and were excluded from data sourcing even when policies were in place to mitigate the economic impacts of the pandemic. Those countries were Kiribati, Marshall Islands, Micronesia, Nauru, Palau, Samoa, Solomon Islands, Tonga, Tuvalu, and Vanuatu.

5. Original legislation, decrees, or policy documents were located for all but four countries.

6. Two countries, Finland and Switzerland, did not have national minimum wages. For Finland, we used the lowest 1% of full-time earnings by occupation from the national statistical office to calculate the effective percentage of the flat rate payment. For Switzerland, we used the wage described as ‘low earners’ by the national statistical office. This wage was below the cap on payments.

7. These countries are Austria, Belgium, Croatia, Denmark, Finland, the Republic of Korea, Luxembourg, and Sweden.

8. These countries are Honduras, Poland, and Sierra Leone. In Honduras, 15 days are available over the course of a year, but no more than 2 days can be taken per month. The Czech Republic also has 9 days of leave available per instance of school or child care center closure with no explicit yearly maximum.

9. In September 2020, paid leave was doubled to 4 weeks.

10. In September 2020, Canada introduced a new program guaranteeing up to 26 weeks of benefits for households that was subsequently extended to 42 weeks.

11. The Republic of Korea introduced additional paid leave for single parents in September 2020 as part of an expansion of the policy. However, while two-parent households received 20 days of paid leave each, single parents were only entitled to 25 days.
